# The “Kots Wrap”: A Technique To Avoid Skin Trauma Related to Isolation Drape Adhesive

**DOI:** 10.7759/cureus.41741

**Published:** 2023-07-11

**Authors:** Joseph T Kots, Linda M Minda, Francis McGeehan, Donna Bennett, Gregory K Deirmengian

**Affiliations:** 1 Orthopaedic Surgery, Thomas Jefferson University Hospital, Philadelphia, USA; 2 Orthopaedic Surgery, Rothman Orthopaedic Institute, Philadelphia, USA

**Keywords:** hip and knee replacement, patient health safety, patient safety culture, patient safety improvement, safety patient

## Abstract

Patients of advanced age or medical diagnoses such as venous insufficiency are predisposed to skin tears when healthcare professionals handle the skin or apply adhesive to it. Isolation drapes are sometimes used to define the area of skin for sterilization prior to a procedure. Such drapes are designed to be secured to the skin with an adhesive edge. Removing such drapes at the end of the procedure can lead to skin tears, especially for patients at risk. We describe a simple technique known as the "Kots Wrap" for applying such an isolation drape in a manner that avoids applying the adhesive edge of the drape to the skin and also allows for removal of the drape in an efficient and atraumatic manner. The use of this technique may help minimize the risk of skin tears in this context and promotes a culture of patient safety.

## Introduction

Prior to the sterilization of a surgical site, nonsterile isolation drapes are often used to define the surgical field and to cover areas that may introduce the potential for contamination. For total hip and knee arthroplasty, at our institution, an isolation drape is used to isolate the foot from the rest of the lower extremity in order to minimize the risk of contamination of the surgical site from its microbial flora. The role of this drape is not to seal the foot from the rest of the field, but rather to cover the foot in an attempt to avoid gross contamination. In the past, the adhesive edge of a 10x10 drape is secured to the skin around the distal leg and the remainder of the drape has been wrapped around the foot and then secured externally with elastic tape.

In some cases, the skin around the distal leg may be subject to injury. Examples include patients with moderate to severe venous insufficiency and elderly patients with thin and friable skin [1,2]. In such cases, the use of adhesive on the skin may predispose the skin to injury at the time of drape removal. We describe a technique for securing an isolation drape to the foot in a manner that minimizes the risk of skin injury at the time of drape removal.

## Technical report

**Figure 1:**
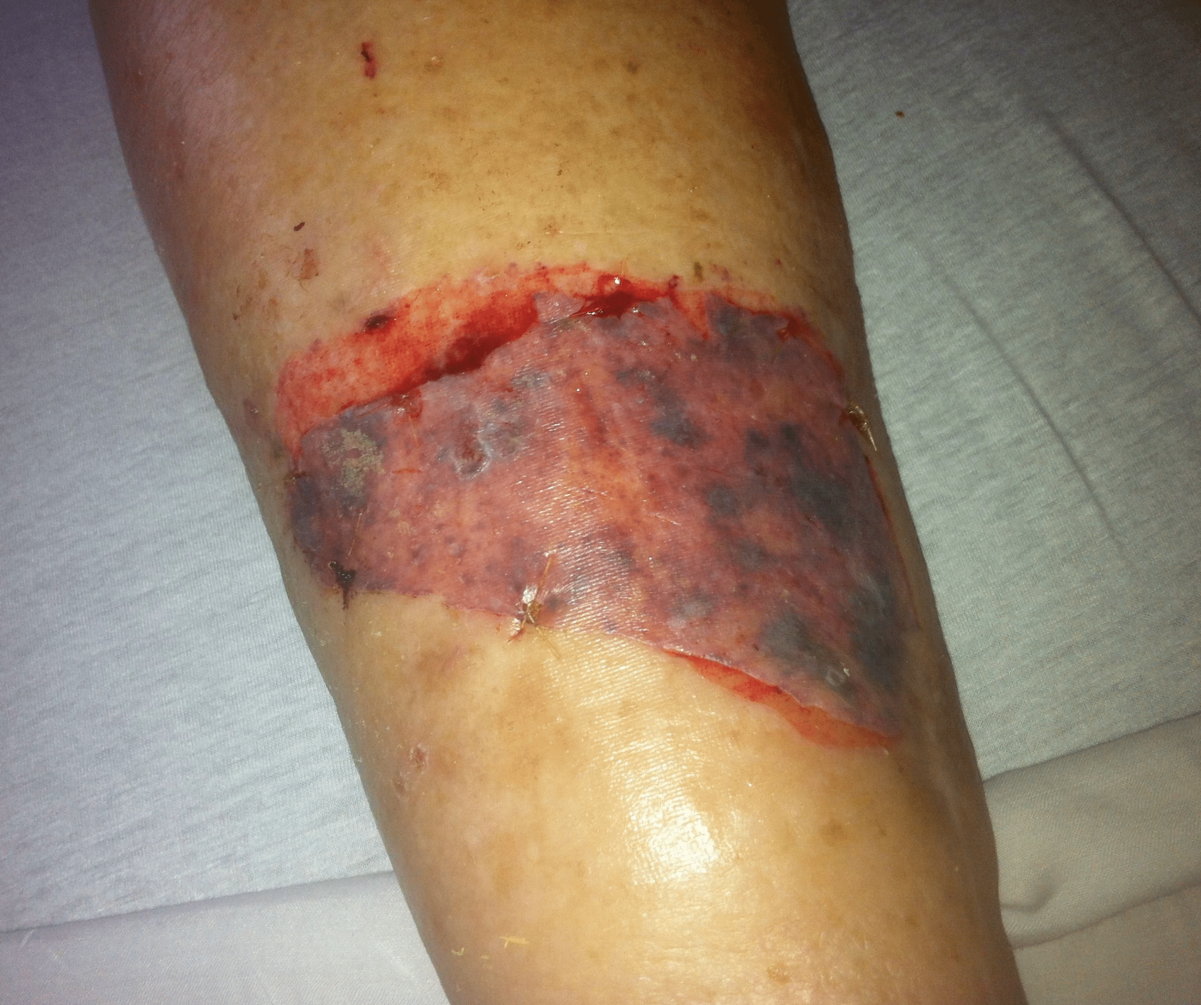
Skin trauma sustained at the time of removal of an isolation drape that was stuck directly to the skin with its adhesive

In order to avoid skin trauma at the time of drape removal (Figure 1), the isolation drape is applied in a manner that avoids applying the adhesive directly to the patients' skin and also in a manner that allows for easy removal of the drape at the end of the case without risking skin damage with the use of scissors. We describe the “Kots Wrap” that achieves these goals.

**Figure 2:**
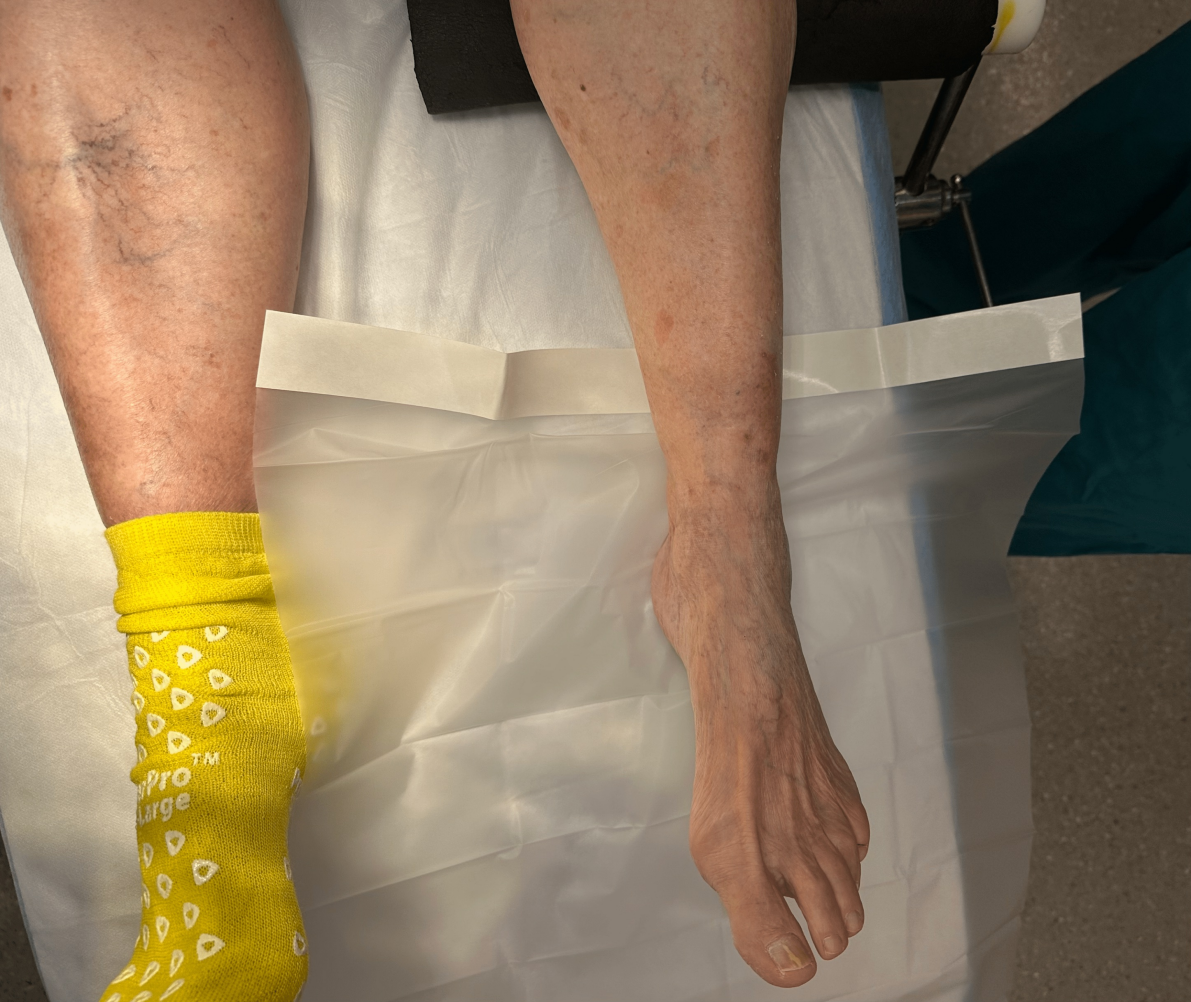
Step 1

Step 1: The isolation drape is placed under the distal leg with the covered adhesive side of the drape oriented toward the skin (Figure 2).

**Figure 3:**
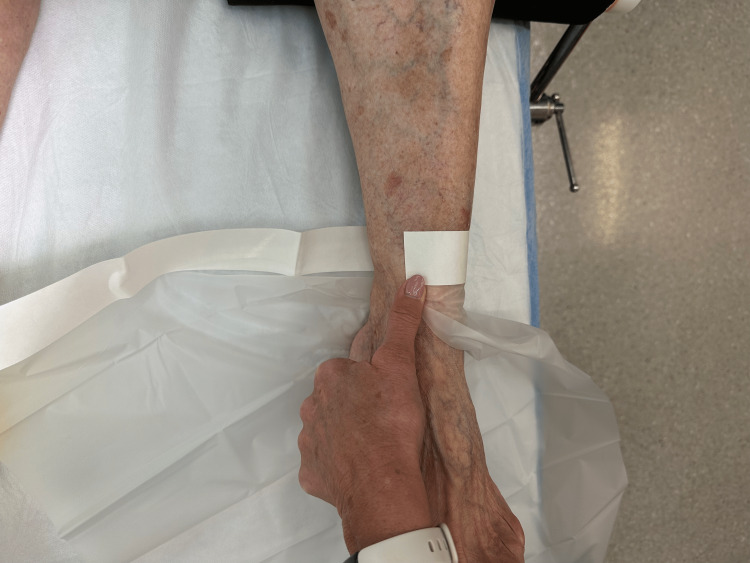
Step 2

Step 2: One side of the adhesive cover is wrapped around the leg to estimate the length of the cover needed to span the leg circumferentially (Figure 3).

**Figure 4:**
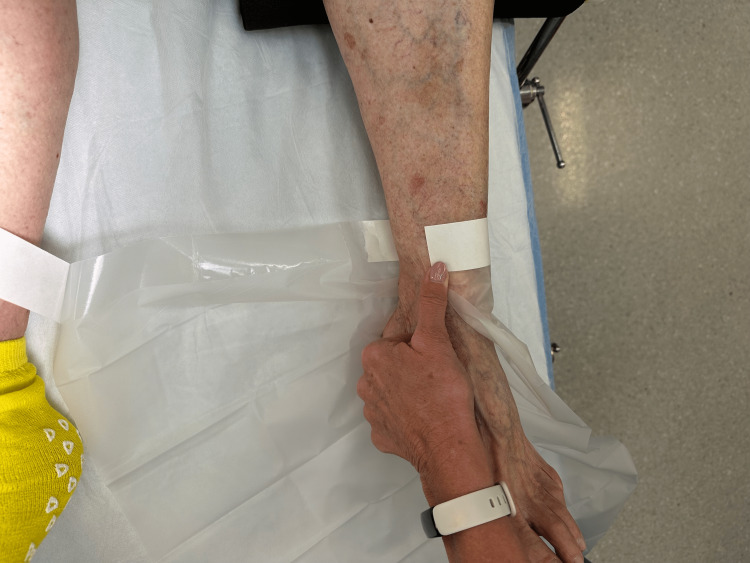
Step 3

Step 3: The cover of the adhesive is torn and removed to expose the adhesive from the remainder of the drape (Figure 4).

Step 4: The exposed adhesive is wrapped around the leg in a snug but not constricting manner and is stuck to the plastic of the drape on the opposite side of the remaining adhesive cover.

**Figure 5:**
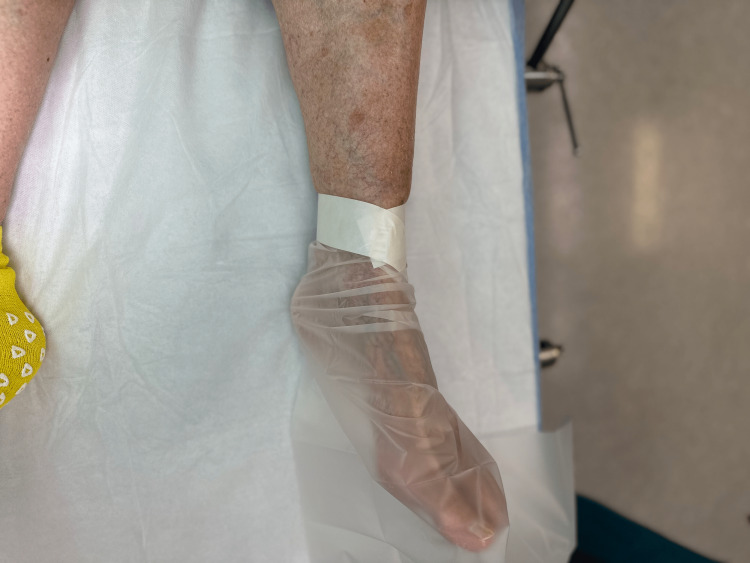
Step 4

**Figure 6:**
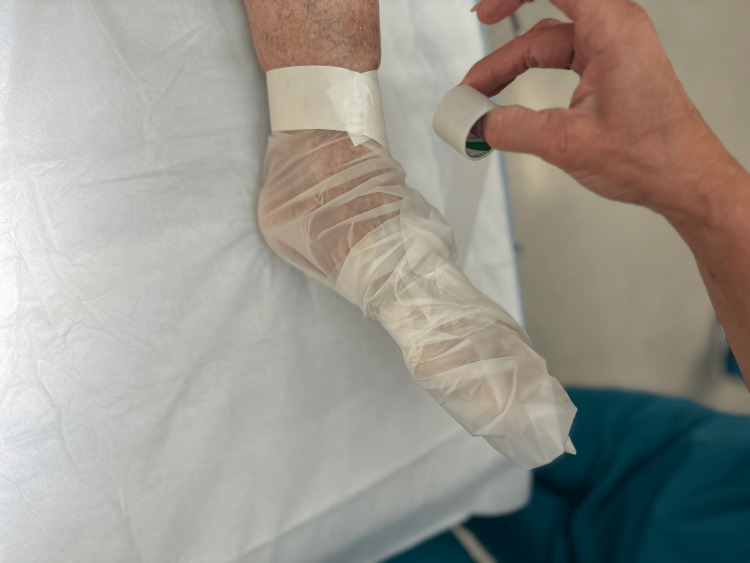
Step 5

Step 5: The part of the isolation drape that surrounds the foot is wrapped around the foot, and elastic tape is wrapped around the outside of the drape for several revolutions to secure the drape to the foot. The tape is applied in a snug but not constricting manner and is restricted to the forefoot and midfoot regions (Figure 6).

At the end of the case, the cuff of the isolation drape is gently torn through the remaining adhesive covering and the entire isolation drape with the overlying elastic adhesive is gently slid off of the foot.

## Discussion

In a surgical procedure that involves an extremely high number of technical steps, each step introduces a risk of complications. Patients with compromised or otherwise friable skin are at risk of skin injuries during handling the skin to manipulate that region of the body or in securing and subsequently removing drapes [3]. In rare cases of patients with compromised skin around the surgical field, the decision may be made to avoid any adhesive application. This decision must be weighed against the potential benefits of securing disposable sterile adhesive surgical drapes to create a seal. Consideration should be given to avoid the use of adherent plastic adhesive incision drapes for the prophylaxis of postoperative surgical site infections as they may not be necessary for orthopedic surgery [4].

The skin around the distal leg is often thin and friable in patients of advanced age or with diagnoses that may compromise skin quality, such as venous insufficiency [5]. At our institution, isolation drapes have been used in order to cover the foot and prevent surgical site contamination from its microbial flora. Such isolation drapes are designed with an adhesive edge to allow for securing the drape to the skin. This introduces the potential for skin damage at the time of removal for patients with skin that is friable. We report a technique of securing the isolation drape around the foot in a manner that eliminates the application of the adhesive to the skin and also allows for the removal of the isolation drape in an efficient manner that may help avoid undue skin damage. We hope that our technique will help avoid undue skin trauma and promote a culture of patient safety.

## Conclusions

We report a technique (the "Kots Wrap") that allows for the application and removal of an isolation drape around an extremity in a manner that could help avoid undue skin tears.
