# Correction: The “Kots Wrap”: A Technique To Avoid Skin Trauma Related to Isolation Drape Adhesive

**DOI:** 10.7759/cureus.c278

**Published:** 2025-08-28

**Authors:** Joseph T Kots, Linda M Minda, Francis McGeehan, Donna Bennett, Gregory K Deirmengian

**Affiliations:** 1 Orthopaedic Surgery, Thomas Jefferson University Hospital, Philadelphia, USA; 2 Orthopaedic Surgery, Rothman Orthopaedic Institute, Philadelphia, USA

The Ethics Statement and Conflict of Interest Disclosures section of this article has been corrected to indicate that this study did involve human subjects.

